# Timing of cardiovascular magnetic resonance imaging after acute myocardial infarction: effect on estimates of infarct characteristics and prediction of late ventricular remodelling

**DOI:** 10.1186/1532-429X-13-S1-M9

**Published:** 2011-02-02

**Authors:** Adam N Mather, Timothy A Fairbairn, Nigel J Artis, John P Greenwood, Sven Plein

**Affiliations:** 1 University of Leeds, Leeds, UK

## Introduction

Cardiovascular Magnetic Resonance (CMR) permits a comprehensive assessment of infarct characteristics following acute myocardial infarction (AMI). The pathophysiological remodelling processes associated with AMI evolve over time and as such, the optimal acute imaging time point to predict medium-term surrogates for outcome has not been established.

## Purpose

This study aimed to define the evolution of myocardial oedema, haemorrhage, microvascular obstruction (MVO) and infarct size by CMR, post-AMI. Secondly, we aimed to assess whether CMR data acquired at ‘day 2’ post-AMI are stronger predictors of infarct size and left ventricular (LV) function, measured at 3 months follow up, than data acquired at ‘1 week’.

## Methods

Fifty-seven patients were recruited with first presentation ST elevation myocardial infarction (STEMI) treated successfully with primary percutaneous coronary intervention. Cine, T2- weighted and late gadolinium enhancement CMR imaging were performed at days 2, 7, 30 and 90 after index presentation.

## Results

Infarct size and extent of myocardial oedema decreased significantly between ‘day 2’ and ‘1 week’ (mean %LV-scar (SD) 27.2 (13.9) vs. 21.6 (14.1), p<0.001 and %LV-AAR (Area At Risk) (SD), 37.9 (15.2) vs. 32.3 (14.3), p=0.003). These changes were accompanied by a significant improvement in LV ejection fraction (%LVEF (SD), 41.7 (9.6) vs. 44.6 (10.1), p<0.001). CMR data acquired at ‘1 week’ were better predictors of LVEF and infarct size at ‘3 months’ than data collected at ‘day 2’. The myocardial salvage index (MSI) ((AAR- infarct size)/ AAR) did not change significantly between ‘day 2’ and ‘1 week’ (%MSI (SD), 27.6 (23) vs. 28.9 (23.8), p=0.85).

## Conclusions

LVEF, infarct size and myocardial oedema change significantly during the first week after AMI. Overall, CMR measurements acquired after one week have greater predictive value for infarct size and LV function at 3 months than data acquired at ‘day 2’.

**Figure 1:**
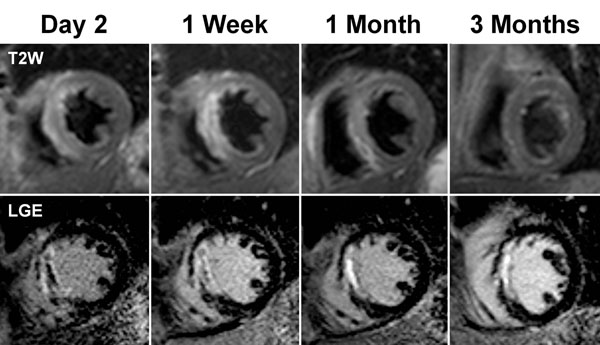
CMR images from a 63 years old male who presented with an anterior ST elevation myocardial infarction. Early after reperfusion, T2W images demonstrated increased signal intensity (myocardial oedema) in the interventricular septum. However, the extent of oedema gradually decreased and by 3 months was undetectable. LGE images from the same patient demonstrated almost transmural infarction in the septum. MVO is present at ‘day 2’ and ‘1 week’ after reperfusion (dark core within the infarct zone) but is undetectable at ‘1 month’. The infarct size decreased during the first month after reperfusion.

